# Protein interaction network of alternatively spliced NudCD1 isoforms

**DOI:** 10.1038/s41598-017-13441-w

**Published:** 2017-10-11

**Authors:** Patrick Asselin-Mullen, Anaïs Chauvin, Marie-Line Dubois, Romain Drissi, Dominique Lévesque, François-Michel Boisvert

**Affiliations:** Department of Anatomy and Cell Biology, Université de Sherbrooke, 3201 Jean-Mignault, Sherbrooke, Québec, J1E 4K8 Canada

## Abstract

NudCD1, also known as CML66 or OVA66, is a protein initially identified as overexpressed in patients with chronic myelogenous leukemia. The mRNA of NudCD1 is expressed in heart and testis of normal tissues, and is overexpressed in several cancers. Previous studies have shown that the expression level of the protein correlates with tumoral phenotype, possibly interacting upstream of the Insulin Growth Factor - 1 Receptor (IGF-1R). The gene encoding the NudCD1 protein consists of 12 exons that can be alternative spliced, leading to the expression of three different isoforms. These isoforms possess a common region of 492 amino acids in their C-terminus region and have an isoform specific N-terminus. To determine the distinct function of each isoforms, we have localised the isoforms within the cells using immunofluorescence microscopy and used a quantitative proteomics approach (SILAC) to identify specific protein interaction partners for each isoforms. Localization studies showed a different subcellular distribution for the different isoforms, with the first isoform being nuclear, while the other two isoforms have distinct cytoplasmic and nuclear location. We found that the different NudCD1 isoforms have unique interacting partners, with the first isoform binding to a putative RNA helicase named DHX15 involved in mRNA splicing.

## Introduction

**Figure 1:**
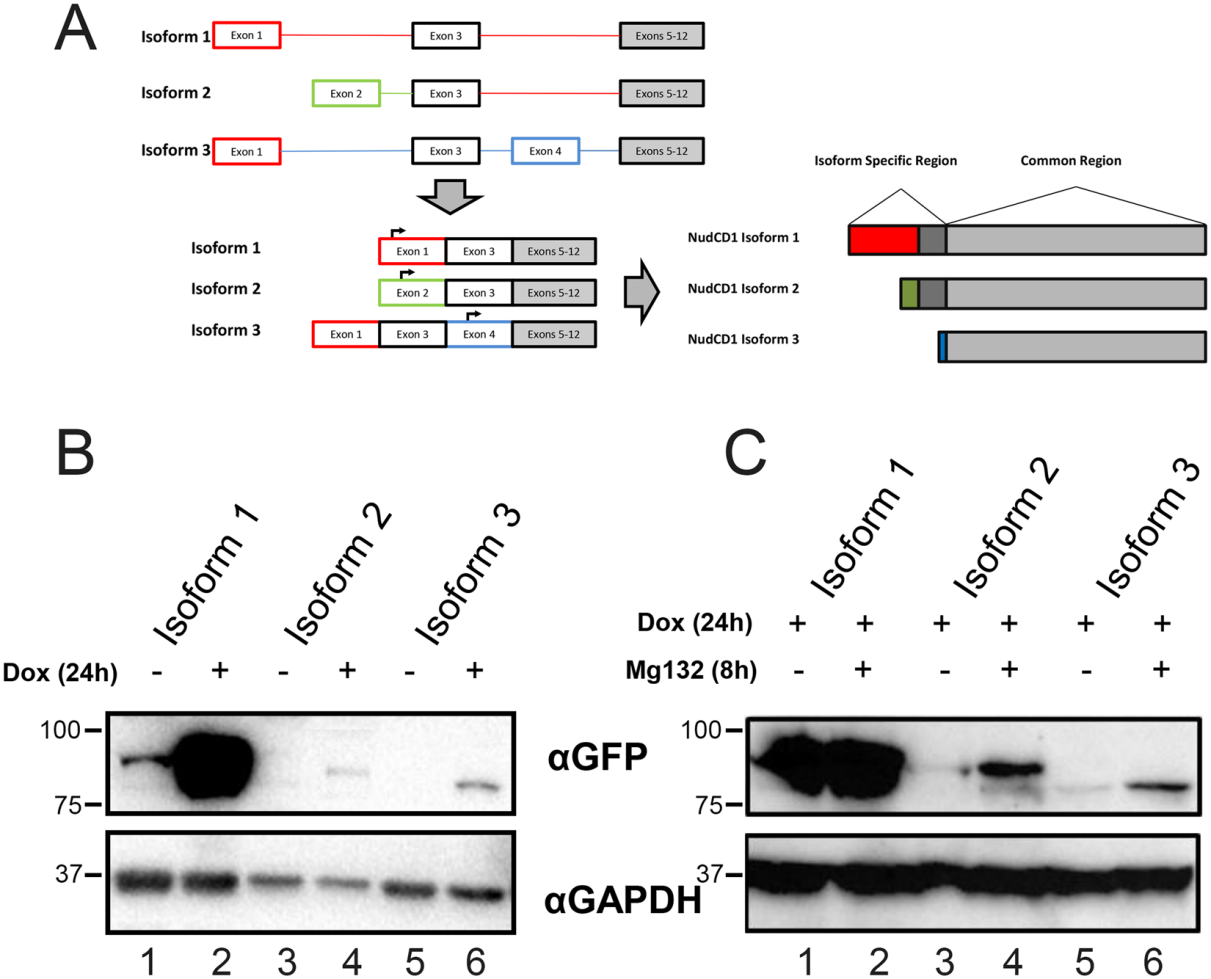
Alternative splicing results in three different NudCD1 isoforms. (**A**) Schematic representation of the first 4 exons of NudCD1 and the resulting isoforms that differs in their N-termimus, while all isoforms include exons 5 to 12. Isoform 1 consists of exons 1 and 3, isoform 2 consists of exons 2 and 3, while the isoform 3 includes the exons 1, 3 and 4, but uses an initiation codon in the fourth exon, resulting in a smaller protein. (**B**) Total protein lysates from U2OS FT cells with doxycycline-induced GFP-tagged NudCD1 isoforms were analyzed by Western blotting using a GFP antibody to confirm expression of the different isoforms following induction using doxycycline (lane 2, 4, 6). (**C**) Total protein lysates from U2OS FT cells with doxycycline-induced GFP-tagged NudCD1 isoforms treated or not with MG132 were analyzed by Western blotting using a GFP antibody.

The nuclear distribution gene C (NudC) protein family is composed of four conserved proteins: NudC, NudC-like (NudCL), NudC-like 2 (NudCL2) and NudC domain containing 1 (NudCD1)^1^, the later also called chronic myelogenous leukaemia 66 (CML66)^2^ or Ovarian cancer-associated antigen 66 (OVA66)^3^. These proteins share a conserved p23 domain conferring them a chaperone activity for binding to p23 and/or Heat shock protein 90 (Hsp90) client proteins^4^. It has been showed that NudC proteins play multiple roles in cell cycle progression, neuronal migration, inflammatory response, platelet production, carcinogenesis^5–8^ and their expression is generally higher in proliferating cells^9^. Among this family, NudCD1/CML66 is the more distant family member and has the least characterized mechanism of action. NudCD1 is a tumour associated antigen highly expressed in human leukaemia, some solid tumours and tumour cell lines^2,10^. Alternative splicing (Fig. 1A) of the mRNA results in three different isoforms sharing a common C terminus (66 kDa isoform 1 [583 aa], 64 kDa isoform 2 [554 aa]^2^, and 61 kDa isoform. While these proteins are often expressed in different cancer cells and tumors, their expression in normal tissues is restricted to testis^10,11^. It also has been demonstrated that NudCD1 was broadly immunogenic, notably following the discovery of specific antibody in 18 to 38% of sera from patients with lung, melanoma and prostate cancers^12,13^.

Knock-down of NudCD1 results in an inhibition of cell proliferation, migration and invasion through regulation of the IGF-1R-MAPK pathway^10,12^, underlining the potential as a target for immunotherapeutic approaches in a variety of solid tumours. Using a high throughput assay to characterize the chaperone-cochaperone interaction network in human cells, Taipale *et al*. established that proteins from the NudC family specifically associate with structurally related β-propeller folds which include proteins such as Coat Protein I (COPI) complex proteins, DEAD/DEAH box helicases and proteins with other functions^14^. COPI complex proteins are involved in cellular trafficking, and modulate autophagy and cell death in cancer cells^15^ and DEAD/DEAH box helicases are involved in almost all aspects of nucleic acid transactions^16^. Furthermore, NudCD1 was found associated with proteins involved in the dissociation of U snRNPs from the lariat-intro complex in experiments identifying protein interactions with the human Pre-mRNA processing 43 (hPrp43) and TFIP11, the human ortholog of yeast Prp43^17^, a post-splicing factor^18^.

Alternative splicing of pre-mRNAs is very common in humans and throughout most eukaryotes, occurring in up to 60% of all genes^19^. Indeed, as many as 95% of human genes containing several exons are undergoing alternative splicing^20^, and this level of tissue and functional diversity in higher organisms is thought to be due in part to alternative splicing^21^. Although alternative splicing is generally thought to be responsible for the diversity of gene products expressed from the genome, the complexity of alternative splicing at the proteome level remains to be characterised^22^. Alternative splicing is well documented at the transcript level, but large-scale proteomics experiments have mostly used a one gene, one protein approach to simplify the number of proteins to be analyzed. Most large scale experiments have focused on using antibodies recognizing a region common to the different isoforms, or have chosen the most characterized protein isoform to include in their study of identifying protein interaction or expression. In this article, we characterized the different NudCD1 isoforms, identifying different roles and subcellular distribution for each of the three isoforms.

## Experimental Procedures

### Cell and SILAC culture

U2OS and U2OS Flp-In T-Rex (U2OS FT) cells were grown as adherent cells in Dulbecco’s modified eagle medium (DMEM) supplemented with 10% fetal bovine serum, 100 U/ml penicillin/streptomycin and 2 mM GlutaMax. Additionally, U2OS Flp-In cells are maintained with 100 μg/ml Zeocin and 15 μg/ml Blasticidine-HCl. For culture in SILAC media, DMEM without arginine and lysine (Life Technologies A14431-01) was supplemented with 10% dialyzed fetal bovine serum (Invitrogen, 26400-044), 100 U/ml Penicillin/streptomycin and 2 mM GlutaMax. Isotopic arginine and lysine were added in either light (Arg0, Sigma, A5006; Lys0, Sigma, L5501) or heavy (Arg10, CIL, CNLM-539; Lys8, CIL, CNLM- 291) to a 28 μg/ml and 49 μg/ml final concentrations of arginine and lysine, respectively. To avoid proline to arginine conversion, an excess concentration of 10 μg/ml of L-proline was added to each medium.

### Gateway Technology, Stable Cell Lines Production and mutagenesis

NudCD1 isoforms 1, 2 and 3, as well as DHX15, were amplified by PCR using oligonucleotides with the BP recombination sites *attB* from a cDNA library generated by RT-PCR using an oligo-dT from mRNA isolated from U2OS cells by Trizol (Invitrogen). The BP recombination reaction using the BP Clonase™ (Life Technologies) was realized between the *attB*-containing PCR products and the *attP*-containing donor vector pDONR 221 (Life Technologies) to generate an entry clone. Then, LR recombination reaction was allowed by LR Clonase™ (Life Technologies) between *attL*-containing pDONR 221-NudCD1 isoforms 1, 2 or 3 and *attR*-containing destination vector pgLAP5.2-GFP. This destination vector is a modified version of pgLAP1^23^ to generate an expression clone with GFP at the C-terminal. DHX15 was cloned into a modified pgLAP1 vector with a Myc tag at the N-terminus. U2OS FT stable cell lines expressing inducible GFP-tagged proteins were generated by transfecting pgLAP5.2-GFP plasmids containing the cDNA of interest along with pOG44 (the plasmid expressing the Flp-recombinase) into U2OS FT cells to allow integration at a specific genomic location^24^. U2OS cells were then selected with the addition of 100 μg/ml Hygromycin B and 10 μg/ml Blasticidine-HCl, and protein induction was achieved by adding 2 μg/ml Doxycyclin for 48 hours. For mutagenesis, we performed a PCR on pDEST47 NudCD1-GFP Isoform 1 to mutate the nuclear localization signal (NLS) from amino acids 10–13 (RVKR to AVAA). The oligonucleotides 5′(p)-CTCTGTTGGATCCCCGCTTCGAG-3′ (forward) and 5′(p)-GTGCCGCCACCGCTAGGGAGCAATTAGCCG-3′ (reverse) were used with the iProof enzyme (BioRad).

### Antibodies

The following primary antibodies were used for immunoblotting and immunofluorescence experiments: anti-GFP (mouse monoclonal, Roche #11814460001), anti-GAPDH (rabbit monoclonal, Cell Signalling #14C10), anti-Myc (rabbit, Cell Signalling #71D10) and anti-SF3A3 (mouse monoclonal, Abcam #56823). The following secondary antibodies were used: anti-mouse IgG-HRP (goat polyclonal, Santa Cruz #sc-2005), anti-rabbit IgG-HRP (goat polyclonal, Santa Cruz #sc-2004), Alexa Fluor® 546 goat anti-mouse IgG (Invitrogen #1010044), Alexa Fluor® 546 goat anti-rabbit IgG (Invitrogen #949214), Alexa Fluor® 488 goat anti-mouse IgG (Invitrogen #1008801), Alexa Fluor® 488 goat anti-rabbit IgG (Invitrogen #1024116) and goat polyclonal anti-chicken IgY DyLight® 650 (Abcam #96954).

### Immunofluorescence Microscopy

U2OS FT pgLAP5.2 NudCD1-GFP isoform 1, 2 or 3 were cultured on glass coverslips in six-well plates. 50% confluent cells were then washed twice with PBS and fixed with 1% paraformaldehyde in PBS for 10 min at room temperature. Fixed cells were washed with PBS and permeabilized using 0.5% Triton X-100 in PBS for 10 min. After another wash in PBS, coverslips were incubated with primary antibodies diluted in PBS (rabbit or mouse antibody depending on the target protein) for 1 h at room temperature. Coverslips were washed once in 0.1% Triton X-100 in PBS and twice in PBS. Primary antibodies were detected with Alexa Fluor 488 goat anti-rabbit and/or Alexa Fluor 546 goat anti-mouse diluted in PBS for 1 h at room temperature. Coverslips were washed in 0.1% Triton X-100 in PBS containing 1 µg/ml 4.6-diamidino-2-phenylindole (DAPI) allowing DNA counterstain for 5 min. After wash with PBS, coverslips were mounted on glass slides and dried overnight at 4 °C in the darkness.

### Immunoprecipitation from SILAC Labeled Cells

U2OS FT cells expressing isoform 1, 2 or 3 of NudCD1 were harvested separately by scraping in PBS and pellets were lysed in immunoprecipitation (IP) buffer (1% Triton X-100, 10 mM Tris pH 7.4, 150 mM NaCl) for 10 min on ice. Insoluble material was pelleted by centrifugation for 10 min at 13,000 *g* at 4 °C and supernatants from the three SILAC conditions were combined. Equal amount of proteins were incubated with GFP-trap agarose beads (ChromaTek) for 2 h at 4 °C. Beads were washed with IP buffer then with PBS. Finally they were resuspended in Laemmli sample buffer prior to SDS-PAGE.

### Gel electrophoresis and in-gel digestion

Proteins were reduced in 10 mM DTT, alkylated in 50 mM iodoacetamide and incubated at 95 °C for 5 min in 1X Laemmli buffer. They were then separated by one-dimensional SDS-PAGE (4–12% Bis-Tris Novex mini-gel, Life Technologies) and visualized by Coomassie staining (Simply Blue Safe Stain, Life Technologies). Following extensive washes in water, the gel was cut into slices and subjected to in-gel digestion with 12.5 ng/ml trypsin (Promega) in 20 mM NH_4_HCO_3_. Tryptic peptides were extracted by 1% formic acid, then 100% acetonitrile. Solvent was removed by lyophilization in a speed vacuum centrifuge, and the tryptic peptides were resuspended in 1% formic acid.

### LC-MS/MS

Trypsin digested peptides were loaded and separated onto a Dionex Ultimate 3000 nanoHPLC system. 10 µl of the sample (2 µg) resuspended in 1% (v/v) formic acid was loaded with a constant flow of 4 µl/min onto a trap column (Acclaim PepMap100 C18 column, 0.3 mm id × 5 mm, Dionex Corporation, Sunnyvale, CA). Peptides were then eluted off and loaded onto a PepMap C18 nano column (75 µm × 50 cm, Dionex Corporation) with a linear gradient of 5–35% solvent B (90% acetonitrile with 0.1% formic acid) over a 4 h gradient with a constant flow of 200 nl/min. Peptides were then electrosprayed into an OrbiTrap QExactive mass spectrometer (Thermo Fischer Scientific Inc.) by an EasySpray source. The spray voltage was 2.0 kV and the temperature of the analytical column was 40 °C. Acquisition of the full scan MS survey spectra (m/z 350–1600) in profile mode was performed in the Orbitrap at a resolution of 70,000 using 1,000,000 ions. Peptides selected for fragmentation by collision-induced dissociation were based on the ten highest intensities for the peptide ions from the preview scan. Normalized collision energy was set at 35% and resolution was set at 17,500 for 50,000 ions. Filling times was set to a maximum of 250 ms for the full scans and 60 ms for the MS/MS scans. All unassigned charge states as well as singly, 7 and 8 charged species for the precursor ions were rejected. Additionally, a dynamic exclusion list was set to retain up to 500 entries with a maximum retention time of 40 seconds using a 10 ppm mass window. To improve the mass accuracy of survey scans, the lock mass option was enabled. Data acquisition was done using Xcalibur version 2.2 SP1.48.

### Quantification and Bioinformatics Analysis

Protein identification and quantification were performed using the MaxQuant software package version 1.5.2.8 as described previously^25^ with the protein database from UniProtKB (Homo sapiens, 16/07/2013, 88,354 entries). For protein identification, carbamidomethylation on cysteine was used as a fixed modification and methionine oxidation and protein N-terminal acetylation were used as variable modifications. The enzyme was set to trypsin, with no cleavages on lysine or arginine before a proline, and up to two miscleavages were allowed. The mass tolerance was set at a maximum of 7 ppm for the precursor ions and 20 ppm for the fragment ions. Re-quantification of selected isotopic patterns was allowed to obtain ratios of all SILAC pairs^26^. We set a threshold of 5% for the false discovery rate (FDR) based on the criteria that the number of forward hits identified from the database was at least 20-fold higher than the number proteins identified in a database containing reverse protein sequences. For protein quantification, we set a minimum of 2 peptides identified for each protein. The mass spectrometry data have been deposited to the ProteomeXchange Consortium (http://proteomecentral.proteomexchange.org) via the PRIDE partner repository with the dataset identifier PXD005493.

## Results

### NudCD1 isoforms cloning, expression and localization

There are three NudCD1 isoforms that are generated by alternative splicing of the first four exons (Fig. 1A). Isoform 1 includes exons 1 and 3, while Isoform 2 is the only one to include exon 2 resulting in a different N-terminal compared to exon 1, but retains the common coding region from exon 3.Isoform 3 includes exon 1 and 3, similarly to isoform 1, but also includes exon 4 which has an additional translation start site (see arrow, Fig. 1A). This results in a smaller isoform with a shorter and different N-terminal (Fig. 1A). Isoforms 1 and 2 do not include exon 4. Additional variants on NIH-NCBI-AceView includes the 3 isoforms that we report here (a to c in their variants). Isoform 4 (or variant d) is an incomplete mRNA lacking exons in the 3′ and does not contain a 3′UTR, probably from incomplete sequencing. Because of the presence of exon 4 with the start codon, the resulting cDNA and protein would be identical to Isoform 3, although the mRNA might differ at the 5′ UTR. The unspliced isoforms (variants e to g) have premature stop codons because of introns, which results in proteins of 58 or 59 amino acids. These unspliced mRNA are thus unlikely to be translated into proteins.

**Figure 2:**
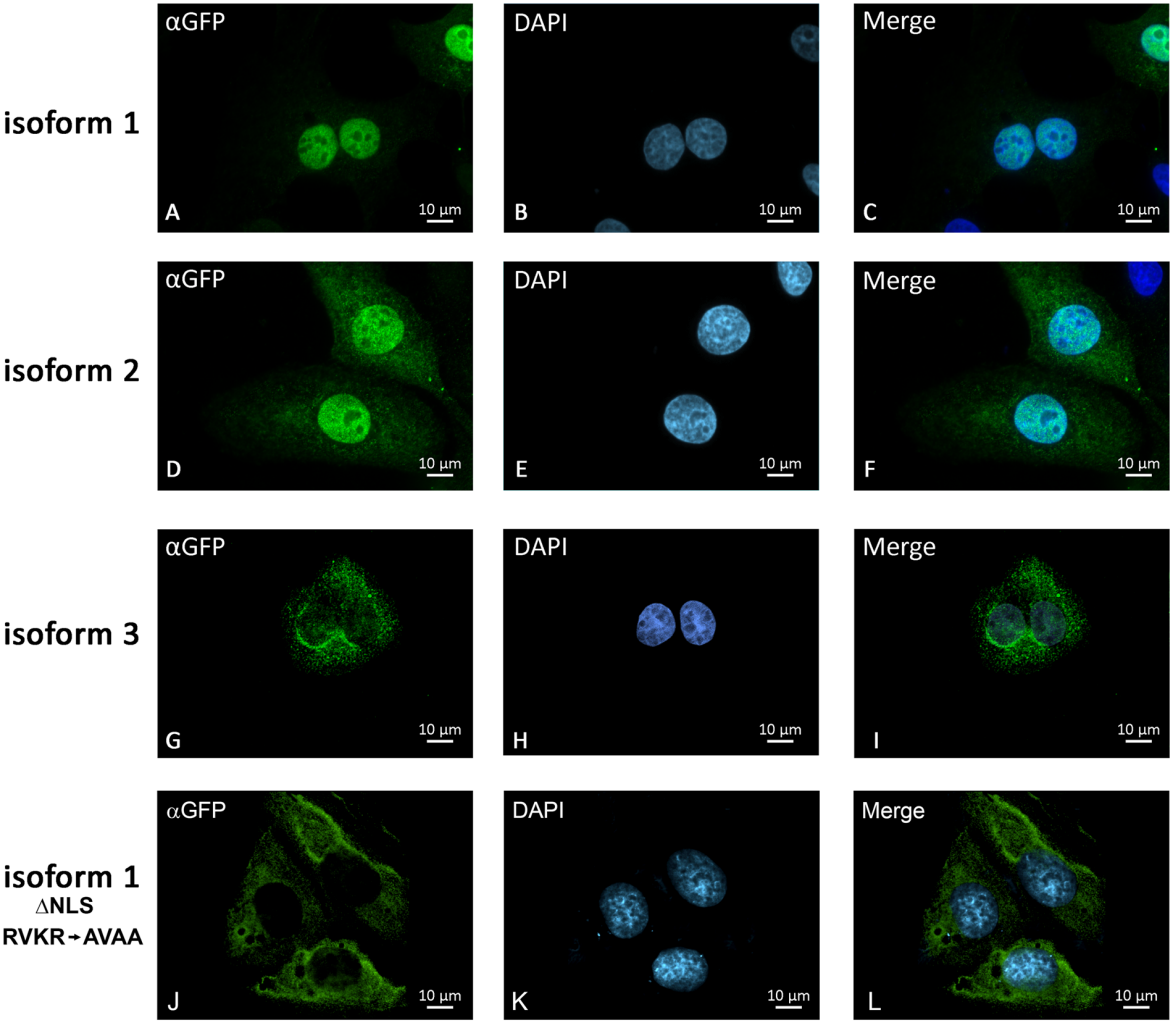
The different NudCD1 protein isoforms have distinct subcellular distribution. Fluorescence microscopy analysis of the U2OS FT cells expressing the respective GFP-fusion proteins was performed using an antibody to GFP (panels A, D and G), and the nuclei were stained with DAPI (panels B, E and H). NudCD1 Isoform 1 shows a strictly nuclear accumulation (**A**), NudCD1 isoform 2 shows both a cytoplasmic and nuclear (**D**) and a predominantly cytoplasmic accumulation is shown for NudCD1 isoform 3 (**G**). Cells transfected with a NudCD1 Isoform 1 with a mutated NLS (RVKR to AVAA) were labelled with an antibody to GFP (**J**), and the nuclei were stained with DAPI (**K**).

Isoform 1 is specifically overexpressed in leukemia and a variety of solid tumor cell lines and is normally detected in testis and heart tissues^2^, isoform 2 is predominantly expressed in testis and weakly expressed in tumor cells^27^ and isoform 3 was identified in a full-length mammalian cDNA project^28^. To determine the functions of each isoforms, we designed fusion proteins composed of NudCD1 (isoform 1, 2 or 3) with GFP at the C-terminus of the protein, since the differences between the isoforms are found at the N-terminus of NudCD1. Three tet-inducible stable cell lines were obtained: U2OS FT pgLAP5.2 NudCD1-GFP isoform 1, 2 and 3. We verified the three NudCD1-GFP isoforms expression in U2OS FT by inducing expression by the addition of 2 µg/ml of doxycycline for 24 h (Fig. 1B). Isoform 1 was strongly expressed compared with isoforms 2 and 3 (Fig. 1B). To determine whether some of the proteins were unstable and rapidly degraded, we induced expression of each isoforms by the addition of doxycycline for 24 hours, and incubated for 8 h with 10 µM Mg132, a proteasome inhibitor (Fig. 1C). The expression of all isoforms was strongly increased following treatment with MG132. The subcellular localization of each isoform was determined by immunofluorescence microscopy experiments (Fig. 2). Expression of each isoforms was induced in U2OS FT pgLAP5.2 NudCD1-GFP cells by the addition of doxycycline for 24 hours, and the cells were fixed prior to immunofluorescence labelling using a GFP antibody, and the nucleus was visualized using DAPI to allow DNA coloration. Imaging of the different isoforms showed a different cellular distribution for each isoform with a predominantly nuclear accumulation of NudCD1 isoform 1 (Fig. 2A–C), cytoplasmic and nuclear accumulation of NudCD1 isoform 2 (Fig. 2D–F) and a mostly cytoplasmic localization of NudCD1 isoform 3 (Fig. 2G–I). These results demonstrate that each isoform of NudCD1 have distinct subcellular distribution.

A nuclear localization signal (NLS) was predicted using PredictNLS^29^ as RVKR in the amino acids 10–13 presents in isoform 1 of NudCD1. To confirm this NLS, we mutated the basic amino acids to alanines (RVKR to AVAA) in our GFP-expressing construct of NudCD1 isoform 1. Cells transfected with NudCD1 isoform1 ΔNLS were fixed prior to immunofluorescence labelling using a GFP antibody, and the nucleus was visualized using DAPI (Fig. 2J–L). Mutation of the putative NLS resulted in a completely cytoplasmic localization.

### NudCD1 isoform-specific interacting partners

**Figure 3:**
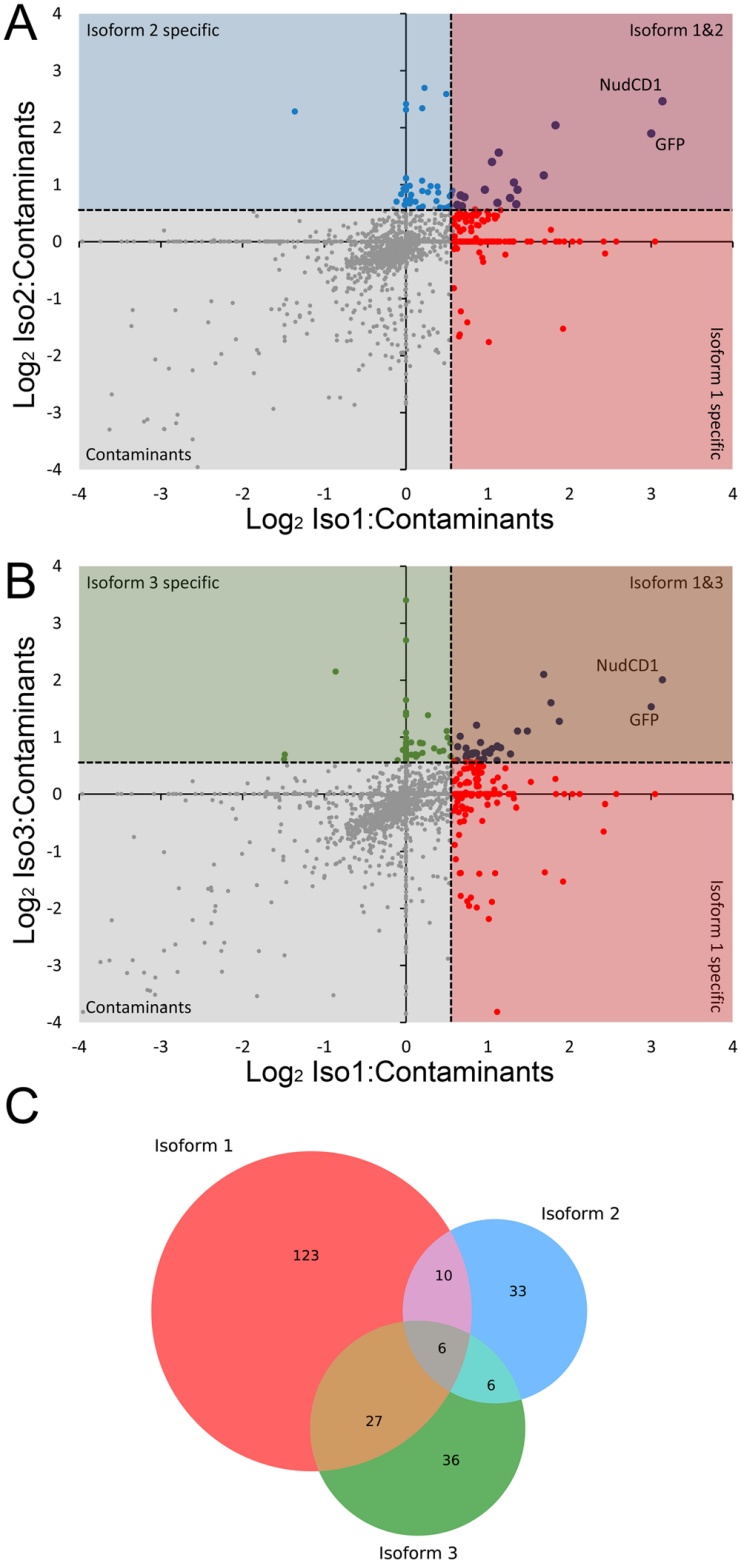
Identification of NudCD1 isoform-specific interacting partners. NudCD1 interacting partners were identified using a combination of immunoprecipitation following by quantitative mass spectrometry analysis using triple-labeling SILAC. Cells not expressing NudCD1 were grown in “light” (L) media, cells expressing NudCD1 isoform 1 were grown in “medium” (M) media and cells expressing either NudCD1 isoform 2 or isoform 3 were grown in cells grown in “heavy” (H) media. Immunoprecipitation were performed using the recombinant GFP-Trap_A reagent by combining a control, isoform 1 and isoform 2 (**A**), or a control, isoform 1 and isoform 3 (**B**). The overlap between the proteins identified as interactors for each isoforms is displayed as a Venn diagram (**C**).

We investigated NudCD1 interacting partners using a combination of immunoprecipitation following by quantitative mass spectrometry analysis using SILAC^30^ to measure differences between the interactions for each isoform, as well as contaminant proteins. Cells not expressing NudCD1 were grown in “light” (L) media and served as a control for nonspecific binding. To identify proteins specifically interacting with each isoforms of NudCD1, cells expressing NudCD1 isoform 1, isoform 2 or isoform 3 were grown in cells grown in “heavy” (H) media. Cells were cultured in SILAC media for at least 6 passages to ensure complete incorporation of the isotopic amino acids, and each experiment were repeated twice (biological replicates). Following lysis, total cell lysates are mixed and subjected to immunoprecipitation using the recombinant GFP-Trap_A reagent^31^ by combining a control and one of the isoform (Fig. 3A,B). These experiments were repeated, and thus represents biological replicates. We identified 162 interacting partners for isoform 1, 53 for isoform 2 and 73 for isoform 3 (ratio IP over contaminant above 1.5 in both replicates, Fig. 3A–C and Supplementary Table 1). By comparing the different isoforms, we found 10 interacting partners specific to isoforms 1 and 2 (Fig. 3A,C), 27 specific to isoforms 1 and 3 (Fig. 3B,C) and 6 specific to isoforms 2 and 3. Four interacting partners were found in common between the three isoforms (Fig. 3C and Supplementary Table 1): FBLN1, FBXO21, LDHB and SRCRB4D.

**Figure 4:**
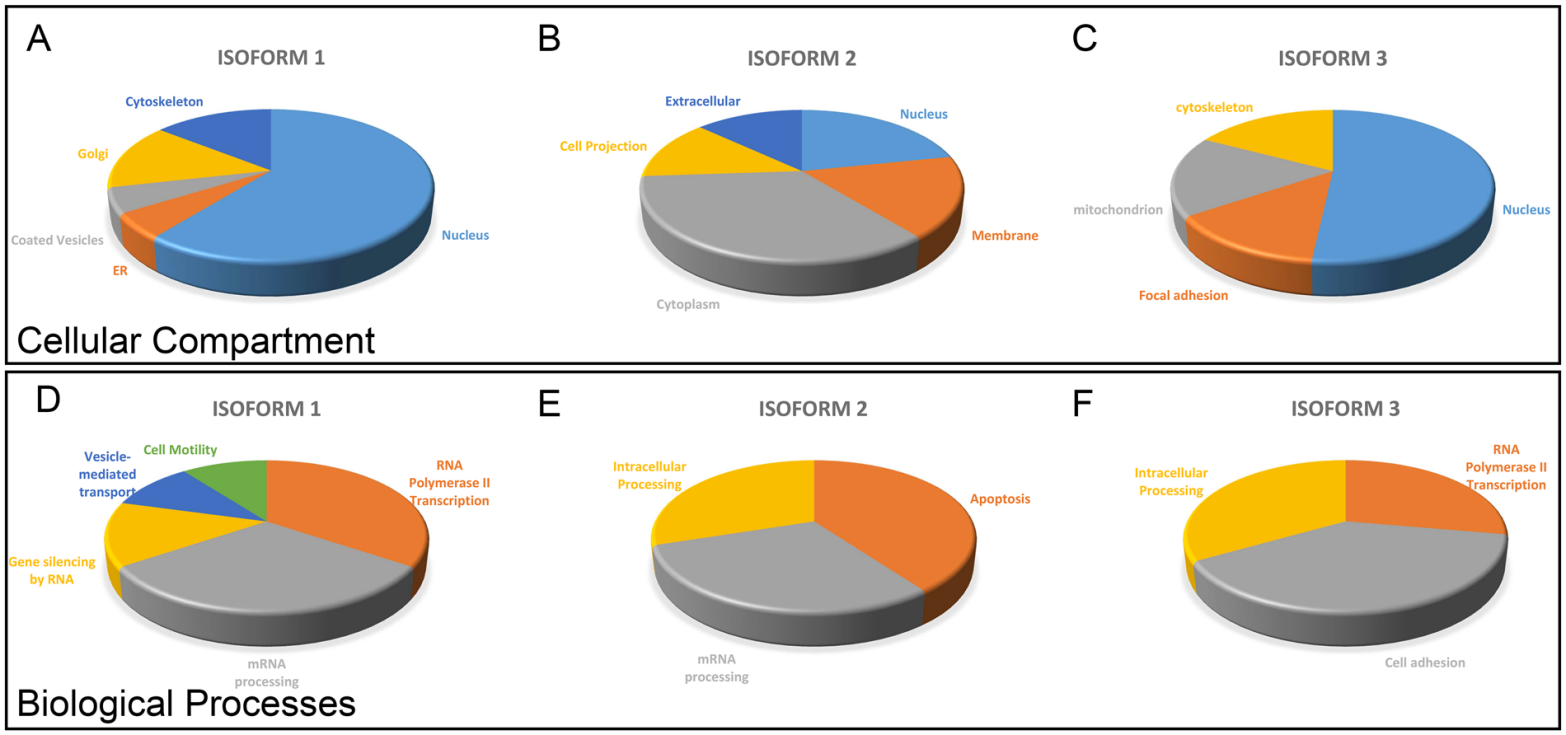
Gene ontology annotations enrichment for each NudCD1 isoforms. The proteins interacting with each of the NudCD1 isoforms were analysed for gene ontology annotations enrichment for cellular compartments (**A–C**) and biological processes (**D–F**) using DAVID.

For each isoform, we classified their partners depending on their cellular compartment localisation (Fig. 4A–C) and biological processes in which they are implicated (Fig. 4D–F), using the gene ontology annotations enrichment tool DAVID^32,33^. A large proportion of NudCD1 isoform 1 interacting partners were nuclear (Fig. 4A), consistent with its subcellular distribution (Fig. 2). Isoform 1 interacting proteins were strongly implicated in biological processes related to mRNA, either RNA polymerase II transcription or mRNA processing (Fig. 4D). A larger proportion of proteins interacting with isoform 2 were cytoplasmic, membrane bound or annotated for cell projection (Fig. 4B). No biological processes appeared to be predominant for isoform 2 with only low scoring functions such as intracellular processes, mRNA processing and apoptosis). Isoform 3 interacted with nuclear proteins, but also included several mitochondrial proteins consistent with a more granular cytoplasmic localization (Figs 2 and 4C).

### NudCD1-DHX15 interaction

**Figure 5:**
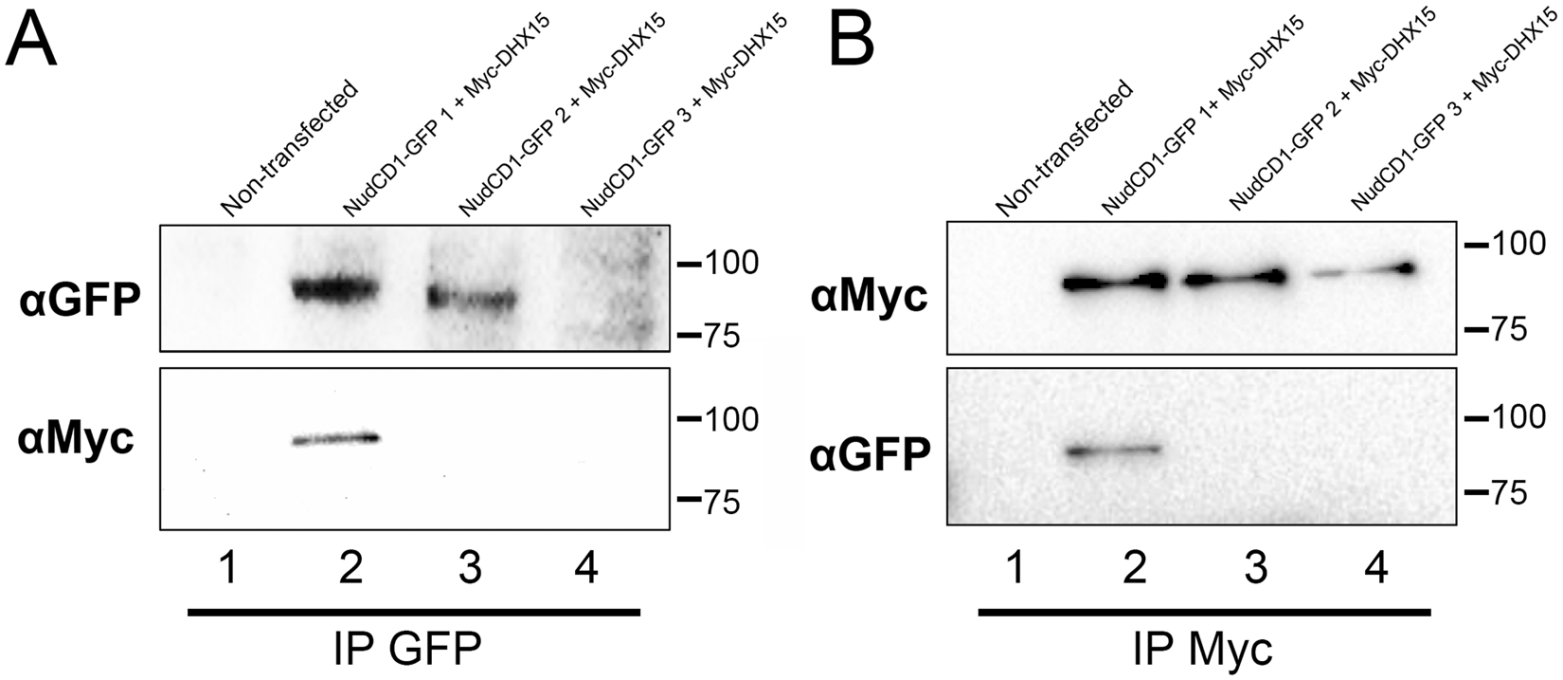
DHX15 interacts with NudCD1 isoform 1 only. To validate the interaction identified by mass spectrometry, co-immunoprecipitation experiments were realized in U2OS FT cell lines expressing NudCD1-GFP isoforms 1, 2 or 3 and co-transfected with a Myc-tagged DHX15. A GFP immunoprecipitation (**A**) and then the reciprocal immunoprecipitation with a Myc antibody (**B**) were performed and revealed with Myc and GFP antibodies.

Of all the proteins identified, DHX15 was found interacting with NudCD1 with the highest enrichment ratio over contaminants, intensity and number of peptides, indicating a strong, near stoichiometric interaction with NudCD1. Interestingly, this interaction was only with the isoform 1, and not isoforms 2 or 3. DHX15 (DEAH-Box Helicase 15) is a putative ATP-dependent RNA helicase implicated in pre-mRNA splicing. To validate the interaction identified by mass spectrometry, co-immunoprecipitation (IP) experiments were realized in U2OS cell lines expressing NudCD1-GFP isoforms 1, 2 or 3 and a Myc-tagged DHX15 (Fig. 5). We have first proceeded with a GFP immunoprecipitation (Fig. 5A) and then the reciprocal immunoprecipitation with a Myc antibody (Fig. 5B) and revealed the NudCD1-DHX15 co-IP with anti-Myc and anti-GFP antibodies. In both co-immunoprecipitations, we could only detect a co-IP between DHX15 and the isoform 1 of NudCD1. No interaction between NudCD1 isoform 2 or 3 and DHX15 was detected. This confirmed the identification of DHX15 as a NudCD1 isoform 1 specific interactor.

**Figure 6:**
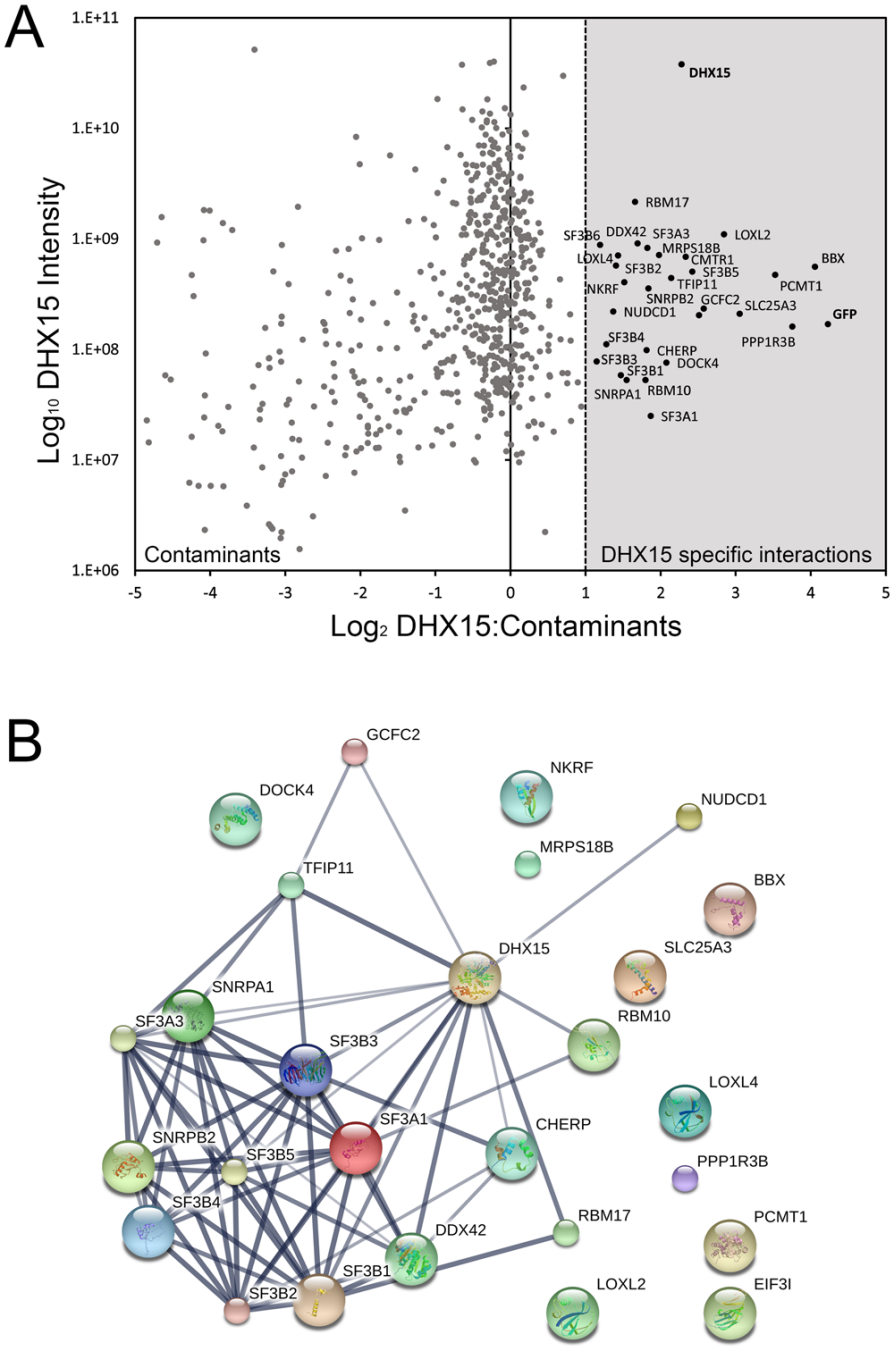
DHX15 interacts with splicing factors from the SF3 complex. (**A**) Control cells were grown in “light” (L) media and cells expressing GFP-DHX15 were grown in “medium” (M) SILAC media. Following lysis, total cell lysates are mixed and subjected to immunoprecipitation using the recombinant GFP-Trap_A reagent. The graph represents the proteins enriched in the immunoprecipitation with a log_2_ of the enrichment ration in the x-axis, and a log_10_ of the intensity in the y-axis. (**B**) A functional protein association networks using STRING shows several known interactions between members of the splicing factor SF3 complex.

To determine whether NudCD1 and DHX15 are part of the same protein complex, DHX15 protein interactions were identified by immunoprecipitation experiments followed by mass spectrometry analysis (Fig. 6A and Supplementary Table 2). Control cells were grown in “light” (L) media and cells expressing GFP-DHX15 were grown in “medium” (M) media. Following lysis, total cell lysates are mixed and subjected to immunoprecipitation using the recombinant GFP-Trap_A reagent as described earlier, by combining the control with the GFP-DHX15 cell lysates. Many interacting partners of DHX15 were identified, including several splicing factors such as RBM10, RBM17, SF3A1 and A3, SF3B1 to B5, GCFC2, TFIP11, SNRPA1 and B2 (Fig. 6A,B). Of these proteins, most of them belonged to the splicing factor 3b protein complex (Fig. 6B), confirming a role in mRNA splicing for DHX15 and suggesting that it may regulate the U2 snRNP binding to the branchpoint sequence in pre-mRNA^34^. While NudCD1 was identified as a DHX15 interacting partners, there are no other overlap between the proteins interacting with NudCD1 isoform 1 and DHX15 interacting proteins, suggesting an exclusive interaction between DHX15 and NudCD1.

**Figure 7:**
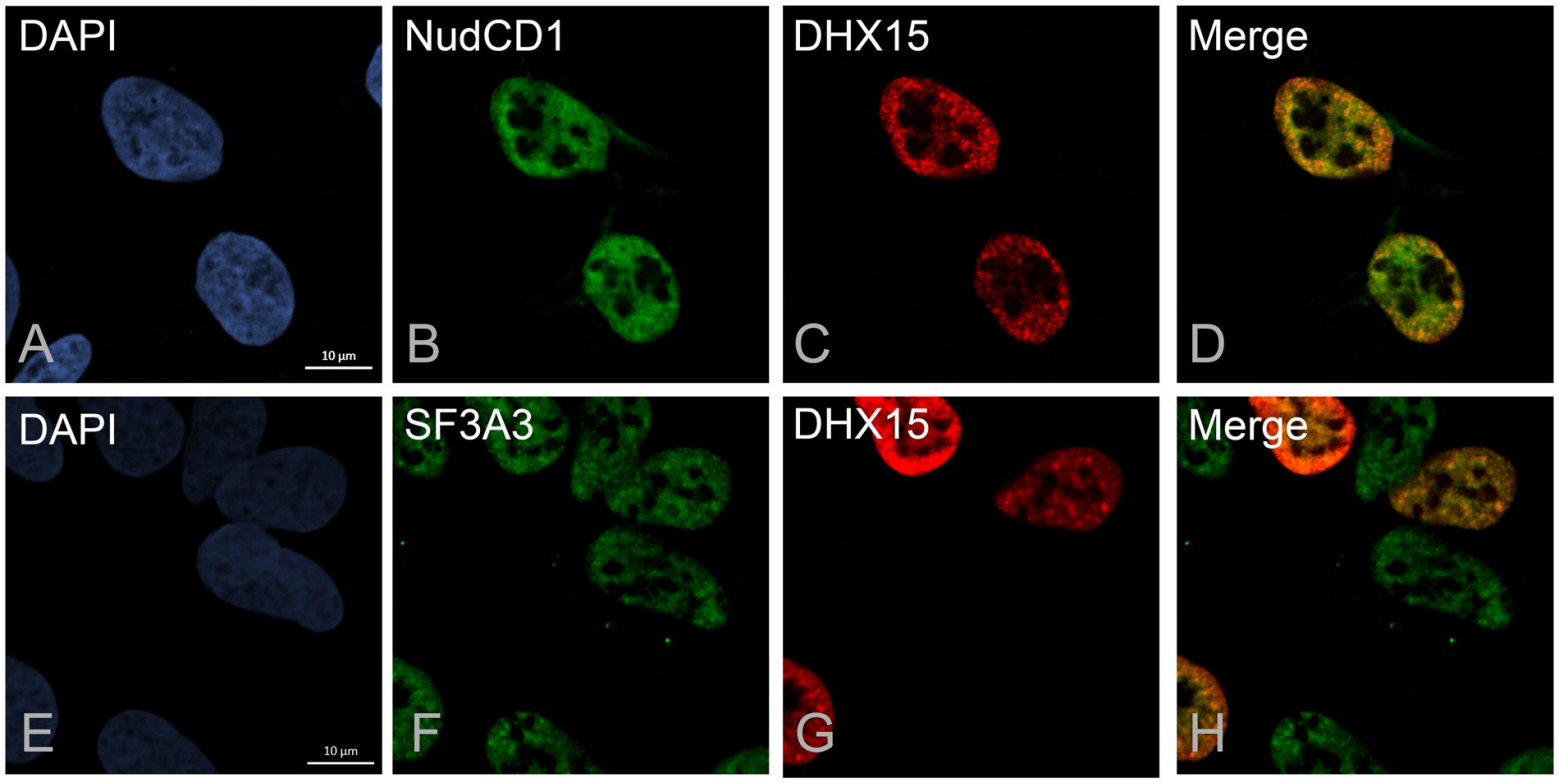
DHX15 colocalizes with SF3 proteins in nuclear speckles, but not NudCD1. U2OS cells transfected with Myc-DHX15 and NudCD1-GFP constructions were fixed and revealed with GFP and Myc antibodies (Fig. 7A–D). To confirm that the localization pattern of DHX15 was consistent with splicing speckles, we also performed immunofluorescence experiments on DHX15 and one of the identified splicing factor, SF3A3 (Fig. 7E–H). The nucleus were stained with DAPI.

Several splicing factors including SF3 proteins localize to nuclear speckles or interchromatin granule clusters^35^. To determine whether NudCD1 and DHX15 interaction was mutually exclusive to the interaction of DHX15 with splicing factors, we investigated whether NudCD1 isoform 1 and DHX15 colocalized in nuclear speckles within the nucleus by immunofluorescence microscopy (Fig. 7). U2OS FT cells transfected with Myc-DHX15 and NudCD1-GFP constructions were fixed and revealed with GFP and Myc antibodies (Fig. 7A–D) allowing the detection of NudCD1 and DHX15, respectively. Both proteins displayed a nuclear localization (Fig. 7B and C). However, while DHX15 localized to structure typical of speckle domains (Fig. 7C), NudCD1 was found mostly diffuse through the nucleus, and excluded from the nucleoli (Fig. 7B). To confirm that the localization pattern of DHX15 was consistent with splicing speckles, we also performed immunofluorescence experiments on DHX15 and one of the identified splicing factor SF3A3 (Fig. 7E–H). The Splicing factor 3A subunit 3 is essential for the formation of the mature 17 S U2 snRNP and the pre-spliceosome assembly^36^. U2OS cells transfected with Myc-DHX15 was also labelled using a SF3A3 antibody (Fig. 7F and G). Cells expressing DHX15 were found to have a similar localization in nuclear speckles as shown by the co-localization with SF3A3 (Fig. 7H). This indicate that NudCD1 does not co-localize with DHX15 in nuclear speckles, consistent with the interaction with NudCD1 in the absence of splicing factors.

## Discussion

NudCD1 was initially cloned from a chronic myelogenous leukaemia cDNA expression library and was found highly expressed in leukaemia and solid tumor cell lines. However, its expression in normal tissues is restricted mainly to testis and heart^2^. It was later found that the NudCD1 gene encodes for three different isoforms that share a common C-terminus (Fig. 1A), and that isoforms 1 and 2 had differential expression resulting from transcriptional regulation: isoform 1 was predominantly expressed in testis and overexpressed in tumor cell lines while isoform 2 was weakly expressed in tumor cell lines^27^. In cells expressing inducible NudCD1 isoforms, we found that the isoform 1 was much more stable compared to isoforms 2 and 3, and that the protein levels increased after inhibition of the proteasome with MG132 (Fig. 1), but not completely. Because all stable cell lines used a unique integration sites, the differences in expression could not be explained by differences at the transcriptional level. This demonstrate that other factors influences the expression and/or the stability of each isoforms in U2OS, as well as in other cells lines such as HCT116 and 293 (data not shown).

Interestingly, each of the three isoforms have distinct cellular localization (Fig. 2), which could explain the large difference in protein stability observed. Isoform 1 is mainly nuclear while isoforms 2 and 3 are found in the cytoplasm, but with a different distribution. Isoform 2 displays a more diffuse cytoplasmic distribution while isoform 3 is found around the nuclear membrane in a punctate pattern, reminiscent of endoplasmic reticulum or the golgi apparatus. It is thus not surprising that each isoforms was found to have few proteins in common in the immunoprecipitation experiments and mass spectrometry analysis (Fig. 3C), indicating that each isoforms are associated with different protein complexes. For isoform 1, the proteins identified were consistent with its nuclear localisation, as most of the proteins identified were involved in RNA polymerase II transcription or mRNA processing (Fig. 4D). For isoform 2, almost a half of the interacting partners identified are proteins found in the cytoplasm, as well as other cellular subcompartments (Fig. 4B). Isoform 3 was found interacting with nuclear proteins, but also with proteins from specific cellular subcompartments such as the mitochondria or focal adhesion points (Fig. 4C). Of all the proteins identified, four were common to the three isoforms (Supplementary Table 1), consistent with the lack of overlap in cellular localisation. Using mass spectrometry and high-throughput LUMIER assays to characterize the chaperone interaction network, Taipale *et al*. also showed that NudCD1 interacts with proteins from the COP complex involved in cellular trafficking, as well as several DEAD/DEAH box helicases^14^, but did not differentiate between the different NudCD1 isoforms. COP complex proteins are involved in cellular trafficking, and appear to modulate autophagy and cell death in cancer cells^15^ and DEAD/DEAH box helicases are involved in almost all the nucleic acid transactions^16^.

In previous studies, it was demonstrated that NudCD1 could play a role in tumorigenesis by increasing cellular proliferation, invasion and metastasis. Downregulation of NudCD1 in tumor cells that showed a high expression of the protein resulted in a down-regulation of some oncogenic genes such as CTSL, MMP15, uPAR, VEGF, COX-2, S100A4, MUC1, MDM2 and RAC1^10^ and an increase of the resistance to 5-fluorouracil-induced apoptosis^11^. Secondly, NudCD1 could promote oncogenesis by enhancing activation of the IGF-1R-ERK1/2 signalling pathway, notably implicated in cell proliferation and differentiation, via its regulation of MDM2 (ubiquitin ligase for IGF-1R)^12^. None of these proteins were found as interacting partners of NudCD1, suggesting that the regulation of the level of these proteins is not through interaction with NudCD1, but could be a consequence of the expression of NudCD1 on other cellular processes that would be involved in regulating the expression of these proteins, such as transcription or splicing.

Of all the proteins identified, DHX15 was found consistently with the highest number of peptides and intensity, suggesting a near stoichiometric association with NudCD1 (Supplementary Table 1). Moreover, this interaction was only observed with the isoform 1 of NudCD1, indicating that the interaction is either occurring only in the nucleus, or that the small difference at the N-terminus of NudCD1 is the region involved in the interaction. DHX15 has been proposed to be involved in mRNA splicing^37^, which was confirmed in our mass spectrometry analysis that identified several splicing factors interacting with DHX15, mostly from the SF3 complex (Fig. 6). DHX15 was identified as the human homolog of the yeast protein Prp43 based on sequence similarities, and could replace the function of Prp43 in a yeast-based splicing assay^38^. Prp43 is involved in spliceosome disassembly via its interaction with Ntr1 and Ntr2 in yeast, through interaction with the N-terminal G-patch domain of Ntr1, resulting in the stimulation of the helicase activity of Prp43 ^39–42^. Ntr1 is also called Tuftelin Interacting Protein 11 (TFIP11) and was identified as an interacting partner of DHX15 in our experiments (Fig. 6). Our experiments have showed co-localization between DHX15 and proteins from the SF3 splicing factor complex that are essential for the formation of the mature 17 S U2 snRNP and pre-spliceosome assembly^36^, further confirming a role for DHX15 in splicing. What is interesting here is that we did not observed any co-localization between NudCD1 and DHX15, and we did not identified any common interacting partners except for the fact that these two protein were found as the highest interacting proteins with each other. This suggest that the interaction is mutually exclusive with the function of DHX15 in mRNA splicing, suggesting a potential role for NudCD1 in regulating or sequestering DHX15 away from splicing factors.

Thus, our study showed a different subcellular distribution for the different isoforms of NudCD1, with the first isoform being nuclear, while the other two isoforms have distinct cytoplasmic and nuclear location. We found that the different NudCD1 isoforms have unique interacting partners, with the first isoform binding to a putative RNA helicase named DHX15 involved in mRNA splicing. These data provide a molecular basis to explain the different contradictory roles observed for NudCD1 in cell proliferation and tumorigenesis upon up or downregulation.

## Electronic supplementary material

Supplementary Information

Supplementary Table 1

Supplementary Table 2
