# Supplementary material for: Protein interaction network of alternatively spliced NudCD1 isoforms
Source: Sci Rep. 2017 Oct 11;7:12987. doi: 10.1038/s41598-017-13441-w (PMC5636827; doi:10.1038/s41598-017-13441-w)
Supplement: Supplementary file 1 — Supplementary Information [file 41598_2017_13441_MOESM1_ESM.pdf]

Protein interaction network of alternatively spliced NudCD1 isoforms.

Patrick Asselin-Mullen, Anaïs Chauvin, Marie-Line Dubois, Romain Drissi, Dominique Lévesque and François-Michel Boisvert

SUPPLEMENTARY TABLES

The following tables provide the data used to generate the graphs from figure 3, 4 and 6:

Supplementary Table 1 - Mass spectrometry analysis of the immunoprecipitation of the different NudCD1 isoforms. The first table includes the protein identification data for the replicate experiments for isoforms 1, 2 and 3. The tables Iso1, Iso2 and Iso3 include the data for the protein identified interacting with each isoforms, including the average ratio of enrichment over the control, as well as the number of peptides identified, the q value and the Andromeda search engine scores. The tables Peptides include all the peptide information for each of the identified proteins, including their ratio of enrichment for each of the replicates.

Supplementary Table 2 - Mass spectrometry analysis of the immunoprecipitation of DHX15. The first table includes the protein identification data for the DHX15 experiments. The table DHX15 includes the data for the protein identified interacting with DHX15, including the average ratio of enrichment over the control, as well as the number of peptides identified, the intensities, the q value and the Andromeda search engine scores.

The complete data, including the raw files and all extracted peptides information can be found at the ProteomeXchange Consortium (http://proteomecentral.proteomexchange.org) via the PRIDE partner repository with the dataset identifier PXD005493.
